# Commentary: Are we over‐pathologising young people's mental health? Locked inside our own building – on disorderism and the need to deflate our language

**DOI:** 10.1111/camh.70028

**Published:** 2025-09-16

**Authors:** Branko M. van Hulst, Maria M. Groen‐Blokhuis, Bram de Ridder, Tycho J. Dekkers

**Affiliations:** ^1^ LUMC Curium, Child and Adolescent Psychiatry Leiden University Medical Centre Leiden the Netherlands; ^2^ Mental Care Group Woerden the Netherlands; ^3^ Arkin Amsterdam the Netherlands; ^4^ Accare Child Study Center Groningen the Netherlands; ^5^ Department of Psychiatry University Medical Center Groningen Groningen the Netherlands; ^6^ Levvel, Child and Adolescent Psychiatry Amsterdam the Netherlands

**Keywords:** Disorderism, pathologising, language, diagnostic labels, decontextualisation

## Abstract

At its core, pathologising is choosing the language of pathology to describe suffering. In youth mental health, the prevailing choice is to use diagnostic labels such as ADHD and autism when describing the problems young people face. A key, yet poorly visible risk of such diagnostic labelling is *disorderism* – a relative neglect of context introduced by the tendency to interpret peoples struggles through the lens of disorders of the individual. At the same time, diagnostic labels serve important functions. Among others, they help families access care and find information, allow researchers to compare findings, and aid policymakers organise funding. Put simply, the functions are too valuable to outright discard, yet the risks too great to ignore. We find ourselves stuck – locked inside our own language. We argue that a path forward lies in recognising the primary way diagnostic labels may cause harm: by sidelining other forms of understanding. Rather than reimagining the labels entirely, a way out of the deadlock could involve profound epistemic humility. If we deflate the labels – removing weight and certainty – we create space for other kinds of understanding.

We shape our buildings; thereafter they shape us.

This famous observation by Churchill applies to architecture, but it has a broader appeal. A recurring question in debates about pathologising youth mental health concerns how the words we use shape our understanding and behavior. Our building might be made out of words rather than bricks, still, the basic principle holds true: we shape our language, thereafter it shapes us.

## On disorderism

At its core, pathologising is choosing the language of pathology to describe suffering. In youth mental health, the prevailing choice is to use diagnostic labels such as ADHD, autism, or depression when describing the problems young people face. We argue that a key, yet poorly visible pattern underlying different risks of such diagnostic labelling is *disorderism* (De Ridder & van Hulst, 2023), which we define as a relative neglect of context, introduced by the tendency to interpret people's struggles through the lens of disorders of the individual.

Take Jason, a fictional 8‐year‐old being treated for ADHD.^1^ He is the youngest in his newly formed class of 34 children. After receiving parent training and medication, he manages to concentrate better and is able to fully take part in class – a successful outcome. Naturally, another child in class now has the weakest concentration. The parents of this child, having seen Jason progress, decide to visit mental health services as well. In its end‐of‐year report, the school concludes that it is capable of giving high‐quality education to classes of over 30 children and that the class size will be maintained. The teacher feels validated in thinking attention problems are best viewed through the lens of ADHD.

Meanwhile, unknowingly and unintentionally, holding younger children back becomes less common, and the school environment has grown a little less accommodating for children with weaker concentration. Note that this is not about inadequate treatment: while guideline‐based care is being provided, our view of what we can expect from an eight‐year‐old quietly shifts. It is the *paradox of successful treatment* – effective individual treatments can lead to a narrowing of what we consider and accept as normal (De Ridder & van Hulst, 2023).

Some often‐mentioned risks of diagnostic labelling can easily be linked to the concept of disorderism and its relative neglect of context (i.e., decontextualisation). The individualisation of social problems, as pointedly articulated by Timimi in the previous issue of *Child and Adolescent Mental Health*, directly feeds into this (Timimi, 2025). But beyond that, decontextualisation has been linked to various risks, such as an overreliance on medication, prognostic pessimism, and stigma (Lebowitz & Appelbaum, 2019). Moreover, when you consider that one's personal context, past and present, forms a large part of one's life story, seeing oneself through the lens of a diagnostic label can undermine a sense of agency in a self‐enforcing manner. Here we arrive at another paradox of diagnostic labelling. We risk zooming out too little, overlooking the societal context of psychological suffering, yet at the same time, we risk zooming in too little, not truly grasping the personal meaning of ‘symptoms’.

## Locked inside our own building

While the debate on the risks of pathologising youth mental health can be polarising, a closer look reveals an area of agreement that is as self‐evident as it is relevant: diagnostic labels serve many different functions for many different stakeholders across a wide range of contexts (Werkhoven, Anderson, & Robeyns, 2022). To give some examples: labels can help families communicate and find information, allow researchers to compare findings across the globe, support clinicians in structuring evidence‐based practice, and aid policymakers in tracking care needs and organise funding. Or, as Sayal and Hiller convincingly convey: at this point in time, diagnostic labels provide us with a way to give people access to evidence‐based care, a legitimisation of their claim for help, a shared language and, with that, acknowledgement and validation of their struggles (Sayal & Hiller, 2025). It is precisely this multitude of functions and stakeholders that poses considerable strain on our ability to discuss the topic without talking at cross‐purposes (Werkhoven et al., 2022).

In addition, as with any classificatory system, diagnostic labels cling to reality in ways that actively shape reality. Once your attention problems are called ADHD, people will react differently, you will act differently – the nature of those problems will have fundamentally changed (Hacking, 2013). To name it is to change it. Complicating matters further, the meaning of diagnostic labels drifts and expands (i.e., concept creep). What we now mean by autism differs from what we meant 20 years ago. As diagnostic labels shape reality and their meaning shifts over time, different risks of diagnostic labelling emerge such as stigma, self‐fulfilling prophecies, and pathologising everyday behaviour.

In all, we have created a language that (1) carries both significant risks and important functions, and (2) is a moving target. Put simply, the functions are too valuable to outright discard, the risks too great to ignore, and the number of stakeholders too vast to truly steer. We find ourselves stuck – locked inside our own language. However, we argue that a path forward lies in recognising the primary way diagnostic labels may cause harm.

## The need to deflate our language

As Rachel Aviv compellingly points out in her book *Strangers to Ourselves* on how we understand ourselves in periods of crisis: “Mental illnesses are often seen as chronic and intractable forces that take over our lives, but I wonder how much the stories we tell about them, especially in the beginning, can shape their course.” Later, Aviv continues “(…) this [psychiatric] framework may also estrange us from the many scales of understanding required, especially in periods of illness or crisis, to maintain a continuous sense of self” (Aviv, 2024).

While fully in line with the main premise that we shape language, which in turn shapes us, Aviv adds something here. She implies that psychiatric language can also cause harm by sidelining other scales of understanding. This way, it is not only about diagnostic labels being a right fit to the problems people experience, it is also about how much room they leave for other perspectives. This is an example of hermeneutical injustice, where psychological language can be so dominant that you are simply not taken seriously when you express your experience in other terms (Mason, 2011). Morten Skovdal gives an excellent example when he describes how his observation – of resourceful children providing home‐based care for very sick parents, developing life skills and forming positive relations with adults in their community – was marginalised by the frame of ‘parentified young carers’ (Skovdal, 2025).

If the core issue is not the diagnostic labels themselves, but rather their dominance in narratives on mental health, then this gives us an alternative route out of the deadlock. Whereas other initiatives seek to completely reimagine labels through more holistic or context‐oriented organising principles (e.g. multi‐axial classificatory systems or complex systems approaches), another approach is to reduce the dominance of the current labels by removing some of their weight and certainty – effectively deflating the language. This epistemic humility might open up space for other kinds of understanding and expressing. Complementing language is already there – in the everyday words people use to share their stories, but also in the language of peer communities (e.g. the recovery movement or the neurodiversity movement), psychotherapeutic traditions, or religious frameworks. Yet, because of their dominant appeal, diagnostic labels seep into these forms of language, gradually displacing them.

How exactly we should deflate diagnostic language is not something we should impose unilaterally – language on youth mental health belongs to all of us. Still, to offer a sense of what deflation might look like, we suggest a few possible directions. First, we could actively communicate that diagnostic labels do not refer to underlying causes, but rather serve as a(n) (optional) starting point for further exploration. Second, we could modify the terms themselves to make them sound less deterministic. For instance, dropping the word disorder (e.g. speaking of ‘attention‐deficit and/or hyperactivity’ instead of ‘attention‐deficit/hyperactivity *disorder*’) might help avoid the false impression that diagnostic labels imply an understanding of underlying causes (van Hulst, Werkhoven, & Durston, 2021). Third, we might reduce the number of categories in future iterations of our diagnostic handbooks as their multitude suggests a level of precision and certainty that is not warranted.

## Neighbors who can enrich our lives

To conclude, we return to the analogy of diagnostic language as a building that shapes us: if we let go of all the extensions and outbuildings, the carports, sheds and ornamental gardens and maybe turn down the music in the main house a bit, space might open up for neighbors who can enrich our lives.

## Conflict of interest statement

TJD serves as a joint editor of *Child and Adolescent Mental Health*. The remaining authors have declared that they have no competing or potential conflicts of interest.

## Funding information

BMH is funded by an Innovations in Youth Care grant from the Netherlands Organization for Health Research and Development (ZonMw). MMG‐B and BR have not received funding related to this commentary. TJD is funded by a VENI grant from the Netherlands Organization for Health Research and Development (ZonMw)/Netherlands Organization for Scientific Research (NWO). He is part of the steering committee of EUNETHYDIS (European ADHD Network), which receives funding from Medice and Takeda for educational purposes.

## Data Availability

Data sharing not applicable to this article as no datasets were generated or analyzed in this commentary.

## Footnotes

^1^
An adapted vignette from De Ridder and van Hulst (2023). Stoornisme: wat het is en waarom het een probleem is [Disorderism: what it is and why it is a problem]. *Tijdschrift voor Psychiatrie, (2023/3)*, 163–166.

## References

[camh70028-bib-0001] Aviv, R. (2024). Strangers to ourselves. Stories of unsettled minds. New York: Vintage.

[camh70028-bib-0002] De Ridder, B. , & van Hulst, B.M. (2023). Disorderism: What it is and why it's a problem. Tijdschrift voor Psychiatrie, 65, 163–166. 36951772

[camh70028-bib-0003] Hacking, I. (2013). Making up people. In Forms of desire (pp. 69–88). Abingdon: Routledge.

[camh70028-bib-0004] Lebowitz, M.S. , & Appelbaum, P.S. (2019). Biomedical explanations of psychopathology and their implications for attitudes and beliefs about mental disorders. Annual Review of Clinical Psychology, 15, 555–577. 10.1146/annurev-clinpsy-050718-095416 PMC6506347 30444641

[camh70028-bib-0005] Mason, R. (2011). Two kinds of unknowing. Hypatia, 26, 294–307.

[camh70028-bib-0006] Sayal, K. , & Hiller, R. (2025). Debate: Are we over‐pathologising young people's mental health? Research shows otherwise: Mental health conditions are not being recognised or diagnosed in healthcare settings. Child and Adolescent Mental Health, 30, 305–307. 40744903 10.1111/camh.70019 PMC12351218

[camh70028-bib-0007] Skovdal, M. (2025). Debate: Are we over‐pathologising young people's mental health? The role of participatory research in moving beyond pathology. Child and Adolescent Mental Health, 30, 299–300. 40677110 10.1111/camh.70023 PMC12351198

[camh70028-bib-0008] Timimi, S. (2025). Debate: Are we over‐pathologising young people's mental health? You'd be in denial if you think we aren't. Child and Adolescent Mental Health, 30, 296–298. 40673361 10.1111/camh.70015

[camh70028-bib-0009] van Hulst, B.M. , Werkhoven, S. , & Durston, S. (2021). We need to rename ADHD: Calling the condition a disorder falsely implies we know of a cause located in the brains of people diagnosed with it—And we don't. Scientific American, 32, 35.

[camh70028-bib-0010] Werkhoven, S. , Anderson, J.H. , & Robeyns, I.A. (2022). Who benefits from diagnostic labels for developmental disorders? Developmental Medicine and Child Neurology, 64, 944–949. 35191027 10.1111/dmcn.15177 PMC9306602

